# Comparative study between intramedullary interlocking nailing and minimally invasive percutaneous plate osteosynthesis for distal tibia extra-articular fractures

**DOI:** 10.1016/j.cjtee.2021.08.004

**Published:** 2021-08-11

**Authors:** Kapil Mani KC, Bandhu Ram Pangeni, Suman Babu Marahatta, Arun Sigdel, Amuda KC

**Affiliations:** a Civil Service Hospital, Kathmandu, Nepal; b Nepalese Army Institute of Health Sciences, Kathmandu, Nepal

**Keywords:** Distal tibia fractures, IMIL nailing, Maiunion, MIPPO technique

## Abstract

**Purpose:**

Treatment of distal tibia fractures poses significant challenge to orthopedic surgeon because of poor blood supply and paucity of soft tissue coverage. There is considerable controversy regarding the superior option of treatment for distal tibia fracture between the minimally invasive percutaneous plate osteosynthesis (MIPPO) technique and intramedullary interlocking (IMIL) nailing for extra-articular distal tibia fractures. The aim of our study is to compare the functional outcome between the two treatment methods.

**Methods:**

This was the prospective comparative study of 100 patients with distal third tibia fractures divided into two groups. The first group of patients were treated with MIPPO technique while the second group of patients were managed by IMIL nailing. Patients were followed up in outpatient department to assess the functional outcomes, malunion, delayed union, nonunion, superficial and deep infection between the two groups. Statistical analyses were performed using the SPSS software (version 16.0).

**Results:**

Average malunion (degrees) in the MIPPO group was 5 (3–7) ± 1.41 *vs.* 10.22 (8–14) ± 2.04 in the IMIL group (*p* = 0.001). Similarly postoperative knee pain in the IMIL group was 10% *vs.* 2% in the MIPPO group (*p* = 0.001). In terms of superficial infection and nonunion, the results were 8% *vs.* 4% and 2% *vs.* 6% for the MIPPO and IMIL group, respectively (*p* = 0.001).

**Conclusion:**

Both procedures have shown the reliable method of fixation for distal extra-articular tibia fractures preserving the soft tissue, bony vascularity and fracture hematoma that provide a favourable biological environment for fracture healing. Considering the results of the study, we have slightly more preference for the MIPPO technique.

## Introduction

Distal tibia fractures classically include the site in the distal tibia between 4 cm and 12 cm from tibia plafond.^1^ Because of its unique anatomical characteristics of subcutaneous location, poor blood supply and paucity of muscular cover anteriorly, the treatment of distal tibia fractures becomes challenging with numbers of complications such as delayed union, nonunion, wound infection, and wound dehiscence.^2^ There are numbers of methods for treatment of distal tibia fractures including open reduction and internal fixation (ORIF) with plating, minimally invasive percutaneous plate osteosynthesis (MIPPO) technique, intramedullary interlocking (IMIL) nailing and external fixation; however optimal management remains controversial.^3^^,^^4^

Even though ORIF with plating provides anatomical reduction and early mobilization, because of extensive soft tissue injury, it cannot be considered as first line option for distal tibia fractures. IMIL nailing technique is another option which avoids soft tissue insult, preserves biological environment around the fracture and provides relative stability to enhance the fracture healing; however high rates of malunion and knee pain cannot be underestimated.^5^^,^^6^ Recently MIPPO has emerged as a good alternative option for distal tibia fractures because it does not disrupt the periosteal tissue and hematoma around fracture, thus maintaining the biological environment and providing a stable biomechanical construct. Nevertheless, it is not free of some shortcomings like wound problems and implant prominence.^7^^,^^8^

The aim of the current study is to compare the functional outcome, the union rate, time to unite the fracture, and various complications between MIPPO technique and IMIL nailing for extra-articular distal tibia fractures.

## Methods

This is a prospective comparative study performed in the department of orthopedics, Civil Service Hospital, Kathmandu, Nepal, from 2012 to 2018. Altogether 100 patients were eligible and operated for study during the 6 years period. After taking permission from the institutional review board, patients were randomly selected using a computer generated plan from the site www.randomization.com (seed no. 22323) to allocate them into two groups, each containing 50 patients. The first group of patients were treated with MIPPO technique while the second group of patients were managed by IMIL nailing.

Patients with OA type A1, A2, or A3 fractures, aged ≥18 years, presence of distal fragment of at least 3 cm in length without articular incongruity, duration of injury <2 weeks, intact neurological and vascular status and patients who met the medical standards for routine elective surgery were included in the study; whereas fractures with intra-articular extension, compound fractures, fractures associated with vascular injuries, poly-trauma patients, those with head injuries and pathological fractures were excluded from the study. Informed consent was obtained from each patient before participation in the study. Sample size was calculated with the assumption of at least of 30% possible difference between the two groups to obtain an alpha error of less than 5% and statistical power of at least 80% and also to include the dropouts.

**Fig. 1:**
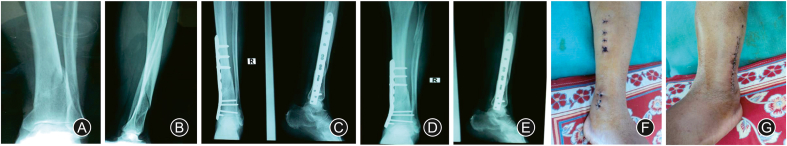
Preoperative (A) anteroposterior and (B) lateral radiographs showing distal third tibia fracture; (C) anteroposterior and lateral views of the ankle and shaft of the tibia showing fracture fixation with MIPPO technique 16 weeks after surgery; (D) anteroposterior view radiograph 20 weeks after surgery; (E) lateral view radiograph 20 weeks after surgery; (F) postoperative multiple healed wound with nonabsorbable suture on tibia side 2 weeks after surgery; (G) postoperative fibular wound 2 weeks after surgery.

**Fig. 2:**
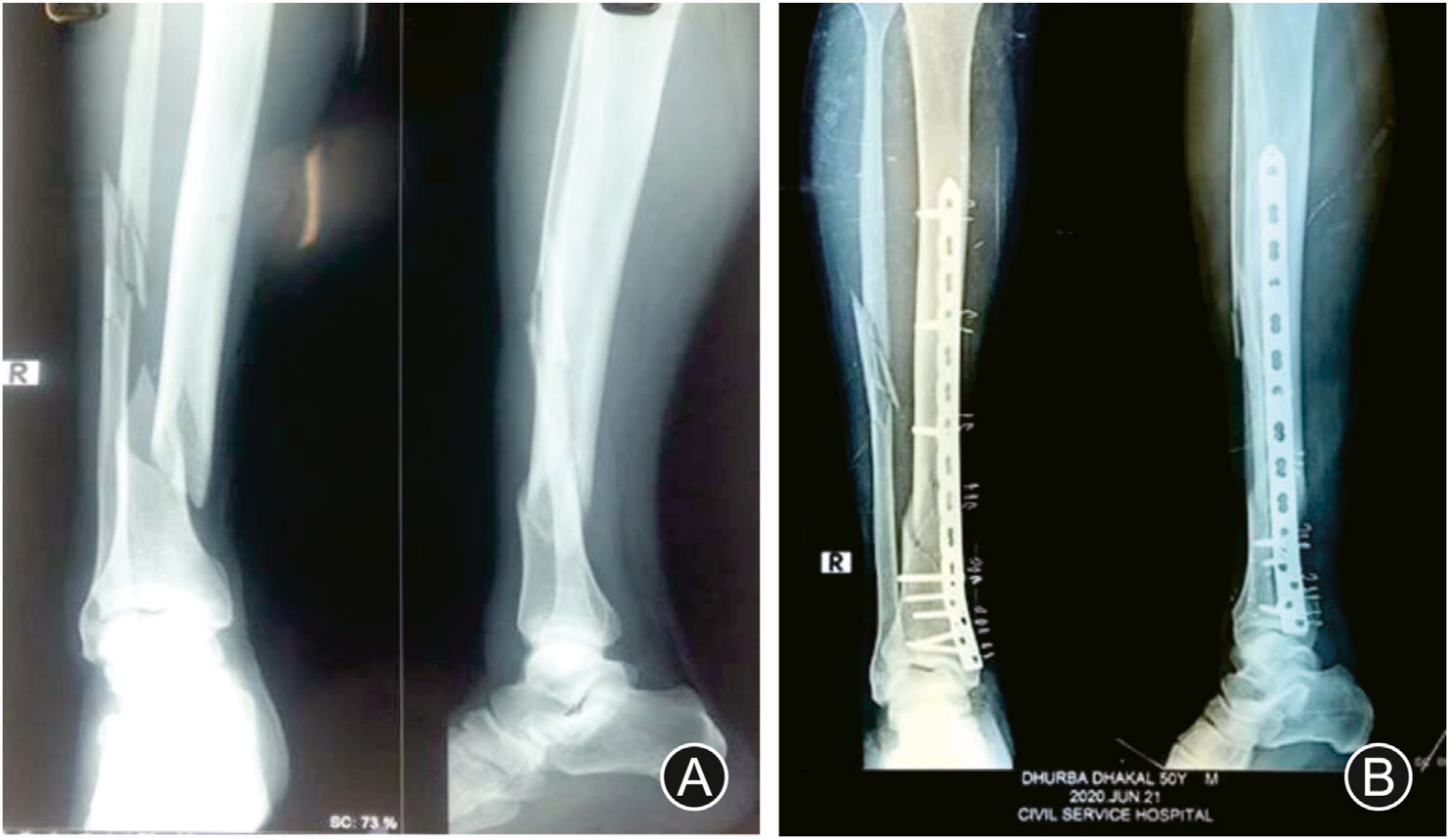
(A) Preoperative anteroposterior and lateral radiographs of distal third tibia and midshaft fibula fractures; (B) Immediate postoperative radiograph anteroposterior and lateral views fixed with precountered locking compression plate with MIPPO technique.

After careful observation and stabilization of injured patient, standard anteroposterior and lateral radiographs of fractured limb with knee and ankle were taken including routine preoperative investigations (Fig. 1. A & B; Fig. 2. A). Meanwhile posterior slab was applied for immobilization of fracture till definitive surgery. Patients with precarious skin condition were managed with limb elevation, regular dressing care and prophylactic intravenous antibiotics. Surgery was delayed till appearance of the ‘wrinkle sign’, but performed within 2 weeks from trauma.

### MIPPO technique

The leg was prepared circumferentially from toes to mid thigh after application of tourniquet. Approximately 3–4 cm longitudinal incision was made over the distal tibia metaphysis preventing the injury of saphenous nerve and vein. Now epiperiosteal tunnel was made either by a blunt retractor or by blunt tip of long plate inserting through the incision towards diaphysis. After traction, manipulation and reduction of fracture, anatomical precontoured plate was positioned on anteromedial aspect of tibia shaft depending on the pattern of fracture. Varus-valgus angulation <5 degrees and anteroposterior angulation <10 degrees and shortening of <10 mm were considered acceptable criteria for reduction. After insertion of plate and achieving the reduction, the plate was temporarily fixed to bone with K-wires and fixed proximal fragment with one locking screw. Distal fragment fixation was completed with combination of cortical nonlocking and locking screws with or without lag screw. Lag screw, if required was inserted either separately or through the nonlocking hole of plate. Fibula was not routinely fixed unless fracture was within syndesmosis region or within 7–10 cm from tibia plafond.

### IMIL nail technique

After application of tourniquet under the same condition above, a vertical incision around 5 cm was made on the anterior aspect of knee from distal tip of patella to proximal aspect of tibial tuberosity. After splitting the patella tendon, entry portal was made in the bare area of tibia extra-articularly around 2 cm proximal to tibial tuberosity. Central position of entry point was confirmed in C-arm with anteroposterior and lateral views. A ball tip guide wire was passed through the entry portal and extended up to the distal end of tibia after provisional reduction of fracture. Intramedullary confirmation of guide wire was confirmed in the C-arm again on both anteroposterior and lateral views. After perfect reduction of fracture, that sometimes requires use of pointed bone holding forceps, sequential reaming was done and fixed with suitable length IMIL nail. The nail was fixed with 2 distal locking screws and 1 or 2 proximal locking screws. Sometimes fracture stability and alignment was enhanced with anteroposterior interlocking screw and/or polar blocking screw.

### Postoperative protocol

Postoperatively, in both groups, fracture was supported with posterior slab for 2 weeks. Standard anteroposterior and lateral views of leg were taken immediately after surgery (Fig. 2B). Active quadriceps and calf strengthening exercises were started next day after surgery. Postoperative intravenous antibiotic was given for 3 days. Wound was inspected on third postoperative day for any sign of wound infection and soakage. Patients were discharged within 1 week if skin condition was satisfactory and wound was dry.

### Follow-up

**Fig. 3:**
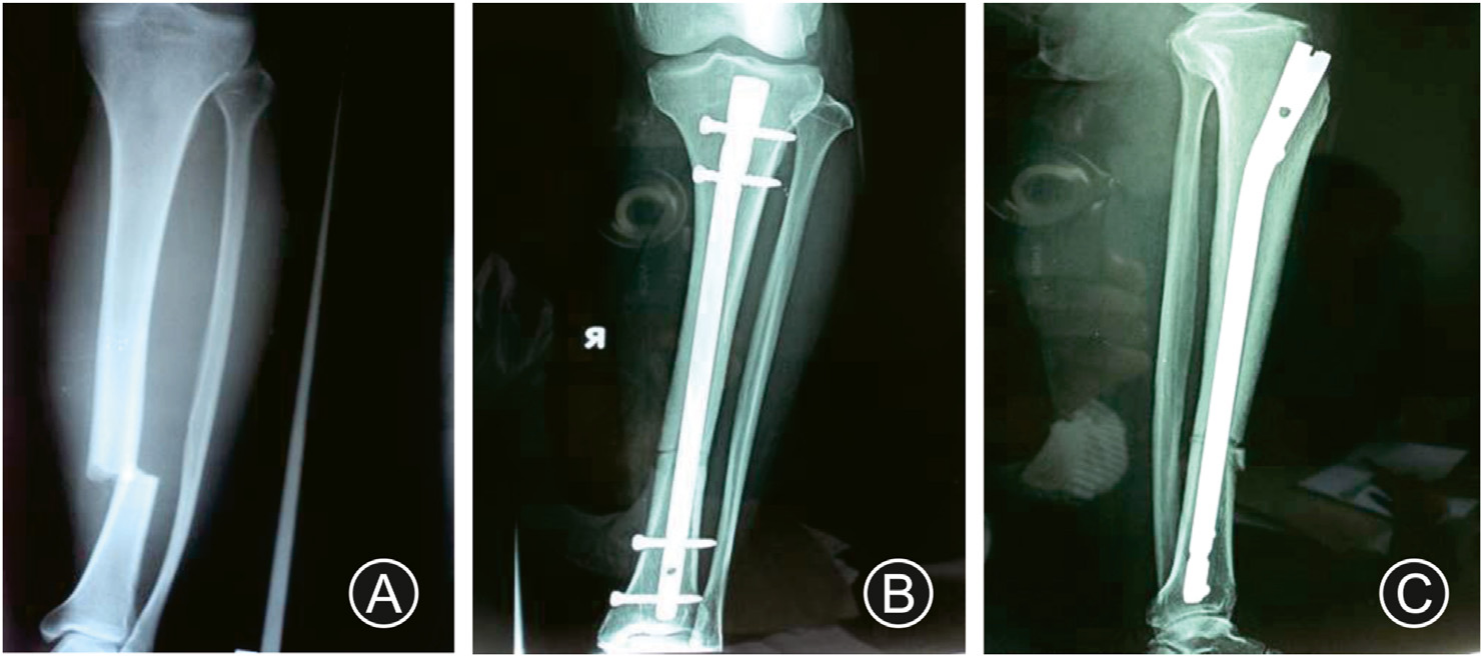
(A) Preoperative radiograph of ankle and leg showing distal third tibia fracture; (B) Postoperative Xray showing IMIL nail 3 months after surgery anteroposterior view; (C) Lateral View.

Suture and posterior slab were removed 2 weeks after surgery. Active range of movements of the knee and ankle joint along with quadriceps strengthening exercises were started. Patients were followed up in outpatient department every 6 weeks for 6 months and then every 3 months thereafter. Post-operative radiograph was performed during each visit in outpatient department (Fig. 1. C-E; Fig. 3B&C). Partial weight bearing was started once callus was visible in radiograph possibly from 6 to 8 weeks. Fracture union was considered when definitive callus was visible in 3 out of 4 quadrants on both anteroposterior and lateral views along with clinical evidence of no pain and mobility on fracture site. If union was not progressed satisfactorily, treatment in the form of bone marrow injection or bone grafting was considered. A clinical evaluation for the functional assessment of the ankle was obtained at 1 year after surgery or once complete union of fracture has occurred using the American orthopedic foot and ankle society (AOFAS) score.

### Statistical analysis

Statistical analyses were performed using the SPSS software (version 16.0). Quantitative variables were documented as mean ± standard deviation. Quantitative variables between the two groups were assessed by independent Student's *t*-test while qualitative data between two groups were assessed by either Chi-square test or Fisher exact test. A *p* < 0.05 was considered statistically significant.

## Results

There were 28 males and 22 females in the first group (MIPPO group) while 26 male and 24 female in the second group (IMIL group). Thirty six fractures were OA type 1, 12 were OA type 2 and 2 were OA type 3 in the first group while 38 patients were OA type 1, 11 were OA type 2 and 1 was OA type 3 in the second group. Average time of surgery in the first group was (59.42 ± 8.97) min while that in second group was (60.80 ± 8.59) min. Fibula fractures were fixed in 18 patients in the first group whereas 20 fibula fractures were operated in the second group. Other parameters like age, time to unite the fracture, surgery time, hospital stay, time to weight bearing, malunion, delayed union, nonunion, superficial infection, deep infection and AOFAS score in both groups are demonstrated in Table 1, Table 2.

**Table 1:** Comparison of the age and treatment outcomes between MIPPO and IMIL nailing groups.

Variables	Group		*p* value
	MIPPO	IMIL nailing	
Age (years)	41.14 (18–61) ±10.52	37.34 (19–68) ±11.21	0.084
Fracture union time (week)	26.06 (19–48) ± 5.35	25.90 (18–46) ± 5.19	0.854
Surgical time (min)	59.42 (40–80) ± 8.97	60.80 (44–80) ± 8.59	
Fluoroscopy time (min)	15.31 ± 1.28	14.12 ± 1.22	
Hospital stay (day)	6.82 (5–10) ± 1.27	6.40 (4–9) ± 1.19	0.092
Time to partial weight bearing (week)	7.32 (6–11) ± 1.49	7.02 (5–10) ± 1.55	
AOFAS score (final follow-up)	84.16 (60–98) ± 8.80	83.84 (61–98) ± 8.87	0.855
Malunion (degrees)	5 (3–7) ± 1.41	10.22 (8–14) ± 2.04	0.001

MIPPO: minimally invasive percutaneous plate osteosynthesis; IMIL: intramedullary interlocking; AOFAS: American Orthopedic Foot and Ankle Society.

**Table 2:** Comparison of delayed union, nonunion, superficial infection, deep infection and knee pain between the two groups, *n* (%).

Variables	Group		*p* value
	MIPPO (*n* = 50)	IMIL nailing (*n* = 50)	
Delayed union	6 (12.0)	7 (14.0)	0.769
Nonunion	1 (2.0)	3 (6.0)	0.04
Superficial infection	4 (8.0)	2 (4.0)	0.04
Knee pain	1 (2.0)	5 (10.0)	0.024
Deep infection	1 (2.0)	1 (2.0)	0.1

MIPPO: minimally invasive percutaneous plate osteosynthesis; IMIL: intramedullary interlocking.

## Discussion

Extra-articular distal third tibia fracture poses significant challenge to orthopedic surgeon because of paucity of soft tissue coverage, peculiar anatomy of distal tibia, significant bony comminution itself. The goal of operative treatment is to restore the anatomical alignment of distal tibia providing a sufficient stability to allow the early mobilization and minimizing the soft tissue and bony devascularization with the hope of decreasing the complications.^2^ For many years, IMIL nailing has been used for management of these fractures because of minimally invasive, less bleeding during surgery, early weight bearing, decreased infection rate, decreased periosteal stripping and faster healing of the fracture. Since distal tibia has wide circular intramedullary cavity with thin cortex as compared to triangular narrow cavity with thick cortex in diaphysis, intramedullary nail which is designed for interference fit in diaphysis cannot provide sufficient stability in distal region.^9^ With development of MIPPO technique that provides the axial and angular stability at screw plate interface rather than plate bone interface and assumed to preserve the periosteal blood supply around the fracture has challenged the IMIL nailing.^2^^,^^10^ Therefore it seems logical to perform the comparative study between these two techniques.

The main outcomes compared were union time, operation time, nonunion, and delayed union, superficial infection, deep infection, malunion, knee pain and AOFAS score. In our study, there was no significant difference in AOFAS, 84.16 (60–98) ± 8.80 *vs.* 83.84 (61–98) ± 8.87; hospital stay (day) 6.82 (5–10) ± 1.27 *vs.* 6.40 (4–9) ± 1.19; fluoroscopy time (min), 15.31 ± 1.28 *vs.* 14.12 ± 1.22; delayed union, 12% *vs.* 14%; deep infection, 2% *vs.* 2%; operation time (min), 59.42 (40–80) ± 8.97 *vs.* 60.80 (44–80) ± 8.59; fracture healing time (day), 26.06 (19–48) ± 5.35 *vs.* 25.90 (18–46) ± 5.19 in MIPPO and IMIL groups.

Average malunion (degrees) in MIPPO group was 5 (3–7) ± 1.41 as compared to IMIL group where average malunion (degrees) was 10.22 (8–14) ± 2.04 which is statistically significant (*p* = 0.001). Similarly postoperative knee pain in IMIL group (10%) was considerably higher than that in MIPPO group (2%) with *p* = 0.001. In terms of superficial infection, the MIPPO group was significantly higher than the IMIL group (8% vs. 4%) with *p* = 0.001. Nonunion in the first group was 2% while nonunion in the second group was 6% which is statistically significant (*p* = 0.001).

Meta-analysis of Hu et al.^1^ demonstrated that both IMIL nailing and MIPPO technique are effective for the distal tibia fractures; however, knee pain and malunion are more common in IMIL group while superficial infection is more common in MIPPO group. In terms of foot function index, IMIL group is superior. Another meta-analysis of Lin et al.^3^ mentioned that IMIL nailing has lower risk of wound complications than does ORIF and MIPPO group. There is no significant difference among three groups in term of nonunion, delayed union and malunion; however they recommended IMIL nailing for distal tibia fractures with better results in relation to wound complications.

There are numbers of other conflicting studies regarding the comparison of IMIL nailing and MIPPO technique. Vallier et al.^11^ reported that IMIL nailing has higher incidence of malunion compared with plate fixation. This may be due to technical and implant related problems like poor quality surgical reduction, inadequate distal locking screws and more distal location of fracture itself. Since anatomical reduction and stable fixation are the effective measure to avoid the malunion of distal tibia fractures, they are achieved better by plating than nailing.^12^ Bong et al.^13^ mentioned that plates have better torsional and bending strain than intramedullary fixation in terms of biomechanics because of spacious medullary cavity of distal tibia and lack of adequate distal locking for intramedullary nail that leads to loss of reduction and malunion. However with the introduction of newer generation nail and adjunctive techniques like angle stable multidirectional distal locking screws as well as polar screws, maintenance of reduction can be better achieved nowadays.^14^ Ali et al.^15^ reported shorter operation time and faster fracture healing time in reamed IMIL nailing compared with MIPPO. Nevertheless, some studies reported that with the development of biologic techniques, plate fixation provides stable fixation and a low rate of infection for distal tibial fractures.^16^

There are some conflicting reports in terms of minimally invasive plating technique as well. Even though MIPPO can restore alignment in meta-diaphyseal region of distal tibia, Collinge et al.^17^ reported the necessity of secondary procedures like bone grafting for delayed union. Other reports also supported above studies with requirement of bone grafting and bone marrow injections in variable frequency for delayed union in MIPPO from 3.8% to as high as 35%.^17^^,^^18^ Hasenboehler et al.^19^ found that MIPPO, even though reliable for distal tibial fractures, can prolong the healing time when it has been used as bridging plate mode. Therefore, inter-fragmentary screw either separately from the plate or through the non-locking hole of plate may prevent the delayed union.

Both the procedures have shown reliable method of fixation for distal extra-articular tibial fractures preserving the soft tissue, bony vascularity and fracture hematoma that provide the favourable biological environment for fracture healing. There are numbers of studies in the literature which are conflicting in terms of superior method of treatment. In our study, even though superficial infection is more common in the MIPPO group, knee pain, malunion and nonunion are significantly more common in the IMIL group. However other parameters like fracture union time, surgery time, fluoroscopy time, hospital stay, time to partial weight bearing and AOFAS score were within comparable ranges. Considering the results of our study, we have slightly more preference for the MIPPO technique.

## Funding

Nil.

## Ethical statement

This study was approved by the Institutional Review Committee of Civil Service Hospital, Kathmandu, Nepal.

## Declaration of competing interest

Authors declare that there is no conflict of interests in our study.
